# Psip1/p52 regulates posterior Hoxa genes through activation of lncRNA *Hottip*

**DOI:** 10.1371/journal.pgen.1006677

**Published:** 2017-04-06

**Authors:** Madapura M. Pradeepa, Fionnuala McKenna, Gillian C. A. Taylor, Hemant Bengani, Graeme R. Grimes, Andrew J. Wood, Shipra Bhatia, Wendy A. Bickmore

**Affiliations:** 1 MRC Human Genetics Unit, MRC Institute of Genetics and Molecular Medicine at University of Edinburgh, Edinburgh, United Kingdom; 2 School of biological sciences, University of Essex, Colchester, United Kingdom; Stanford University School of Medicine, UNITED STATES

## Abstract

Long noncoding RNAs (lncRNAs) have been implicated in various biological functions including the regulation of gene expression, however, the functionality of lncRNAs is not clearly understood and conflicting conclusions have often been reached when comparing different methods to investigate them. Moreover, little is known about the upstream regulation of lncRNAs. Here we show that the short isoform (p52) of a transcriptional co-activator—PC4 and SF2 interacting protein (Psip1), which is known to be involved in linking transcription to RNA processing, specifically regulates the expression of the lncRNA *Hottip–*located at the 5’ end of the Hoxa locus. Using both knockdown and knockout approaches we show that *Hottip* expression is required for activation of the 5’ Hoxa genes (*Hoxa13* and *Hoxa10/11*) and for retaining Mll1 at the 5’ end of Hoxa. Moreover, we demonstrate that artificially inducing *Hottip* expression is sufficient to activate the 5’ Hoxa genes and that *Hottip* RNA binds to the 5’ end of Hoxa. By engineering premature transcription termination, we show that it is the *Hottip* lncRNA molecule itself, not just *Hottip* transcription that is required to maintains active expression of posterior Hox genes. Our data show a direct role for a lncRNA molecule in regulating the expression of developmentally-regulated mRNA genes *in cis*.

## Author summary

Long noncoding RNAs (lncRNAs) have been implicated in various biological functions including regulation of gene expression. However, the mechanism through which they regulate gene expression is not clearly understood. Here we show that a transcriptional co activator—Psip1 specifically regulates the expression of the lncRNA *Hottip*. Moreover, using multiple approaches, including lncRNA depletion, genetic manipulation of Hottip locus, transcriptional activation and premature termination of lncRNA transcript along with RNA localization, we demonstrate that *Hottip* lncRNA regulates expression of neighbouring *Hoxa* genes.

## Introduction

The mammalian genome encodes ~10,000 long noncoding RNAs (lncRNAs)[1]. Although very few of these molecules have been functionally characterised, a small number have been shown to function by binding to various protein complexes to regulate gene expression[2–6]. Some lncRNAs have been reported to affect gene expression *in trans*[7,8], whereas others, such as Kcnq1ot1, Xact, Xist and Tsix, function *in cis* (reviewed in[9]). Other lncRNAs likely function in the cytoplasm through binding to other regulatory RNAs, e.g. miRNAs[10].

It has also been difficult to distinguish whether lncRNA function is conferred by the process of transcription or by the RNA molecule itself. Concerns have been raised with respect to limitations and discrepancies in various methodologies used to study lncRNA function[11–13]. Contrasting conclusions have often been reached when comparing knockdown and knockout studies of lncRNA loci—e.g. *HOTAIR*, *MALAT1* and *Halr* [14–17].

With the exception of relatively well characterized lncRNAs like Xist[18], H19[19,20] and Kcnq1ot1[21,22], many recently described lncRNAs lack genetic evidence to support their function *in vivo*. Indeed, recent efforts to phenotype mouse knockouts for 18 lncRNA genes identified only 5 with strong phenotypes[23]. With the list of lncRNA loci with unknown function increasing, there is a pressing need to rigorously dissect the functional mechanisms of individual lncRNA loci. Additionally, most research has focused on the downstream functions of lncRNAs and, with the exception of lncRNAs involved in imprinting and dosage compensation, little is known about the transcriptional regulation of lncRNAs themselves. Compared to lncRNA sequences, the promoters of lncRNA genes are conserved, and are enriched for homeobox domain containing transcription factor binding sites[24], which suggests lncRNA expression is a regulated process.

**Fig 1:**
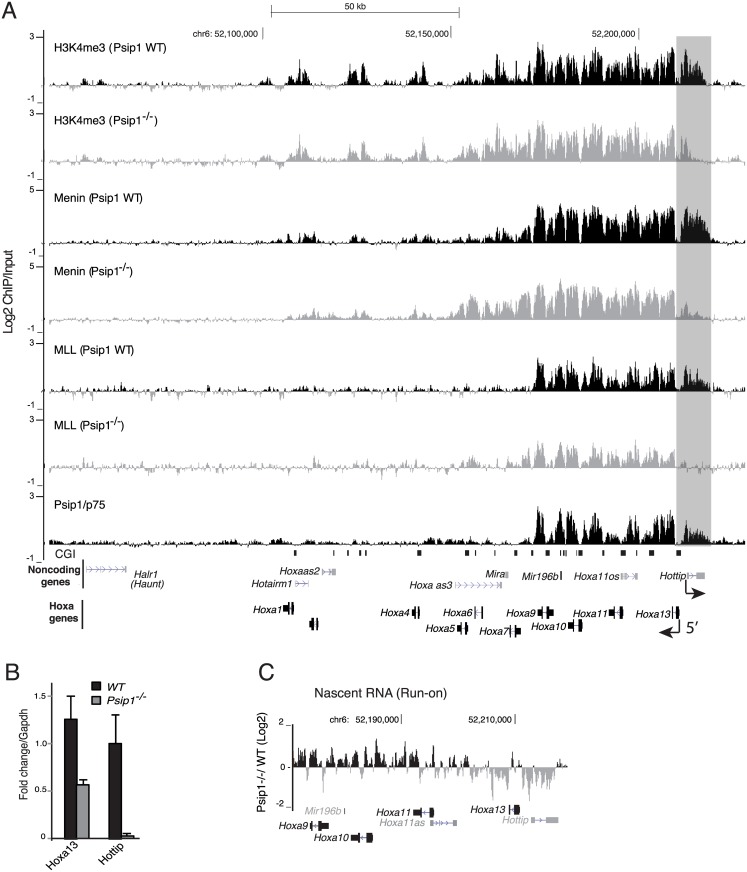
Reduced *Hottip* expression and Mll occupancy in *Psip1*^*–/–*^. (A) Mean Log2 ChIP/input for Psip1/p75, Mll1, Menin and H3K4me3 in WT and *Psip1*^*–/–*^MEFs over *Hoxa* clusters from custom tiling arrays[35]. Annotated noncoding transcripts (grey, top) and Hox gene transcripts (black) are shown below. (n = 2 biological replicates). Genome co-ordinates are from the mm9 assembly of the mouse genome. Direction of transcription for *Hoxa13* and *Hottip* genes are indicated with arrow below. (B) Mean (± s.e.m) expression, assayed by RT-qPCR and normalized to *Gapdh*, of *Hoxa13* and *Hottip* in *WT* and *Psip1*^*–/–*^MEFs, (n = 3 biological replicates). (C) Nimblegen tiling microarray data showing log2 ratio of *Psip1*^*–/–*^/ *WT* run-on transcribed RNA (nascent RNA) over posterior Hoxa genes n = 2 technical replicates.

Mammalian *Hox* loci are important model systems for the investigation of lncRNA functions. Expression of many noncoding RNAs within *Hox* clusters is tissue specific[25–29], and have been linked to the regulation of *Hox* mRNA genes [7,14,30,31]. At the *Hoxa* cluster, the *lncHoxa1/Halr—*also known as *Haunt* is located ~ 50 kb away from 3' end of *HOXA* (Fig 1A) and has been shown to repress *HOXA1* expression *in cis*[32]. Importantly, a recent study demonstrated that *Haunt* lncRNA plays a distinct role as a repressor while its DNA sequence functions as an enhancer for *HOXA* genes[15]. *HOTAIRM*, located between *HOXA1* and *HOXA2*, is expressed antisense to coding *HOXA* genes, and is implicated in retinoic acid induced activation of *HOXA1* and *HOXA4* during myeloid differentiation[33]. *HOTTIP* lncRNA is transcribed in an antisense direction from the 5' end of *HOXA13* (Fig 1A*)*, and is reported to be important for targeting MLL through interaction with WDR5 to maintain posterior (5') HOXA expression in distal tissues[3].

PC4 and SF2 interacting protein (Psip1), also known as LEDGF, has been suggested to play an important role in regulation of Hox genes[34]. We have recently demonstrated the role of the p75 isoform of Psip1 (Psip1/p75) in recruiting an Mll complex to expressed Hox genes[35]. The alternatively spliced short isoform of Psip1 (Psip1/p52) lacks the C-terminal Mll or integrase binding domain (IBD), but shares the chromatin binding PWWP and AT hook like domains at the N-terminus. Psip1/p52 binds to H3K36 trimethylated (H3K36me3) nucleosomes via the PWWP domain and can modulate alternative splicing by recruiting splicing factors to H3K36me3[36].

Here, we show that Psip1/p52, but not Psip1/p75 regulates the expression of the lncRNA *Hottip*, which is located at the 5’ end of the Hoxa locus and transcribed in an antisense direction away from *Hoxa13*. We show that the *Hottip* RNA binds to, and is required for, activation of genes at the 5’ end of Hoxa establishing a firm role for a lncRNA molecule in the regulation of gene expression *in cis*. This also adds a new role for Psip1/p52 in RNA-based processes.

## Results

### Psip1 is required for expression of lncRNA *Hottip*

In mammals, the active state of Hox genes is maintained by Compass-like complexes containing the MLL (Mix lineage leukemia) histone H3K4 methyltransferases. Hox repression is maintained by Polycomb (PcG) complexes[37]. We recently demonstrated that the transcriptional co-activator Psip1/p75 and Mll co-occupy expressed Hox genes, and that loss of Psip1 leads to reduced Mll1 (and Mll2) occupancy at active Hox genes[35]. Most strikingly, at the extreme 5' end of Hoxa, where the *Hottip* lncRNA is located[3], Mll binding is completely lost in *Psip*^*–/–*^MEFs compared to wild type (WT) (Fig 1A). Reduced Mll1 is accompanied by concurrent loss of H3K4me3 and Menin—a common component of Mll1 and Mll2 Compass-like complexes[38]. We noted that absence of Psip1 results in complete loss of expression of the lncRNA *Hottip* and reduced expression of *Hoxa13*, which is located adjacent to *Hottip* at the 5’ end of Hoxa and which has previously been described as one of the target genes of *Hottip* (Fig 1B)[3]. In contrast, other *Hottip* target genes–*Hoxa9*, *a10*, and *a11*[3] are up-regulated in *Psip1*^*–/–*^MEFs despite the loss of *Hottip* expression (Fig 1C)[35]. Nascent run-on transcription analysis shows that these effects occur at the level of transcription (Fig 1C). Together with the binding of Psip1 to the expressed 5’ part of Hoxa (Fig 1A), these results suggest that Psip1 might function as a transcriptional coactivator to regulate expression of the *Hottip* lncRNA.

### Depletion of Psip1/p52 and *Hottip* leads to reduced expression of 5’ Hoxa genes

**Fig 2:**
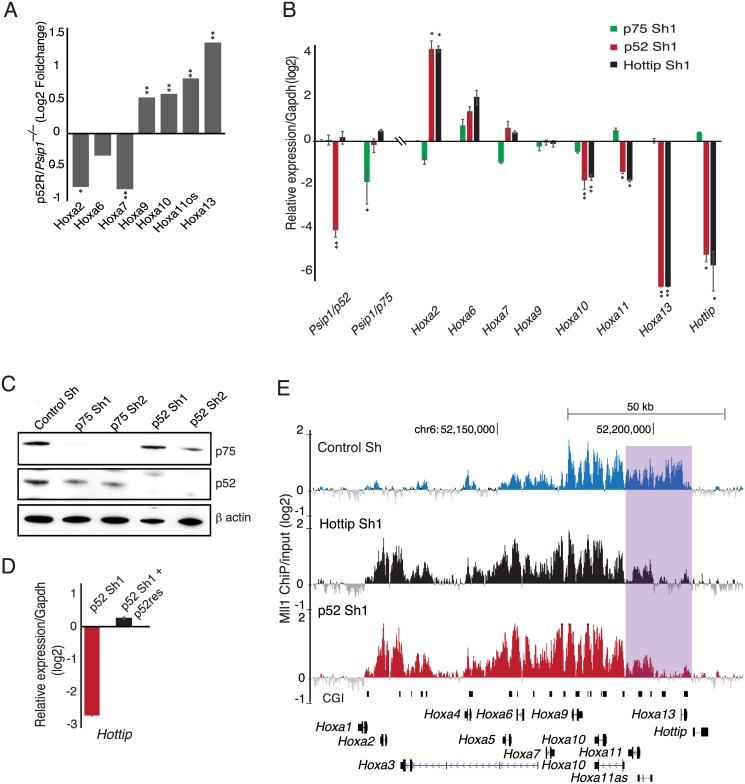
Psip1/p52 and *Hottip* are important for expression of 5’ Hoxa genes. (A) Agilent expression microarray data showing Log2 fold change in expression of Hoxa genes in *Psip1*^*–/–*^MEFs upon rescue with *Psip1/p52* (p52 rescue / *Psip1*^*–/–*^MEFs) (n = 4 biological replicates) * p < 0.05, ** p <0.01. (B) Log2 mean (± s.e.m) relative expression, assayed by RT-qPCR and normalized to *Gapdh*, of Hoxa genes, along with *Psip1/p52*, *Psip1/p75*, and total *Hottip* (exon 2) transcript, in limb cells transduced with shRNAs targeting p52 (red bars, p52 sh1) p75 (green bars, p75 sh1) and *Hottip* (black bars, *Hottip* sh1) relative to cells transduced with a mammalian non-targeting sh RNA (control) (n = 3 biological replicates). * p < 0.05, ** p <0.01. (C) Immunoblotting of limb cells after shRNA knockdown of p52 and p75 Psip1 isoforms with Psip1 antibody (A300-847a) which recognizes both p52 and p75[36]. β-actin served as loading control. Two different sets of shRNAs (sh1 in (a) and sh2 in S1 Fig) were used for knockdown along with a mammalian non-targeting shRNA as control (control_sh). Knockdown of *p52*, *p75* and *Hottip* using independent lentiviral shRNAs (sh2) confirms that mis-regulation of Hox genes is not due to off-target effect of shRNAs (S1B Fig). (D) Mean log2 expression of *Hottip*, in limb cells transduced with sh RNAs targeting p52 (red bars, p52 sh1) and those cells rescued transiently with shRNA resistant p52 cDNA (green bars, p52 sh1 p52res). Fold change in expression was normalized to *Gapdh*, relative to mammalian non-targeting shRNA (control) (n = 3 biological replicates). (E) Mean Log2 ChIP/input across Hoxa cluster for Mll1 from limb cells transduced with control shRNA (Control_Sh), shRNA targeting p52 (p52 sh1) and *Hottip* (*Hottip* sh1) as described in (B) & (C). Genome co-ordinates are from the mm9 assembly of the mouse genome.

Stable rescue of *Psip*^*–/–*^MEFs with the p52 isoform of Psip1 led to an increase in expression of posterior Hoxa genes (Fig 2A) suggesting a role for the short Psip1 isoform in this regulation. To confirm this finding in a different cell type we analysed Psip1-mediated Hoxa expression in a limb bud mesenchymal cell line (14fp) which retains the distal limb-specific expression pattern of posterior or 5' Hox genes[39]. Psip1 is expressed at high levels in the distal limb buds of mouse embryos, where *Hottip* and 5’ Hoxa genes are also highly expressed (S1A Fig)[3]. Moreover, Hoxa13 expression is required for patterning of the distal limb [40].

To identify which isoform of Psip1 regulates *Hottip* in the limb bud cell line we knocked down *Hottip* and also the two separate isoforms of Psip1 using two independent sets of lentiviral shRNAs each specifically targeting the 3' UTR of Psip1/p52, the C-terminus of Psip1/p75 and *Hottip* RNA. Knockdown efficiency was confirmed by RT-qPCR analysis (Fig 2B and S1B Fig) and by immunoblotting for Psip1 isoforms (Fig 2C). Knockdown of Psip1/p75 had no significant affect on *Hottip* or *Hoxa* genes in these cells. However, specific knockdown of Psip1/p52 led to down- regulation of 5’ *Hoxa* genes—*Hoxa13*, *a11* and *a10*, with *Hoxa13* expression being the most strongly abrogated (Fig 2B). Knockdown of p52 also strongly downregulated *Hottip* expression (Fig 2B and 2D) and this was rescued by expression of a shRNA-resistant p52 cDNA (Fig 2D). Knockdown of *Hottip* had an almost identical affect on 5’ Hoxa expression as p52 knockdown (Fig 2B) and is consistent with the reported effects of *HOTTIP* knockdown in human foreskin fibroblasts[3]. These data suggest that it is the p52 isoform of Psip1, not p75, that specifically activates *Hottip* lncRNA transcription. Moreover, these data support an earlier report that the *Hottip* lncRNA is involved in maintaining the active chromatin domain at 5’ Hoxa genes[3].

We found a significant reduction in total *Hottip* RNA levels in the p52 knockdown cells (Fig 2B), which shows that reduced *Hottip* RNA levels are not simply due to the known effect of Psip1/p52 on RNA splicing[36].

### Mll1 occupancy over Hoxa cluster is altered upon *p52* & *Hottip* knockdown

It has been suggested that *Hottip* has a role in maintaining an MLL complex through interaction with the WDR5 component[3]. Consistent with this, ChIP showed that Mll1 occupancy was significantly reduced across posterior Hoxa genes in limb bud cells upon knockdown of *p52* or *Hottip* compared to control knockdown (Fig 2E), Intriguingly, whilst Mll1 was completely lost from *Hoxa13* upon depletion of p52 and *Hottip* (Fig 2E), it was gained at 3' Hoxa genes (*Hoxa1* –*a6*), concomitant with the increase in expression of these 3' Hoxa genes upon p52 or *Hottip* depletion (Fig 2B). This redistribution of Mll is consistent with the redistribution of H3K4me3 and Menin across Hoxa and *Hottip* loci in *Psip1*^*–/–*^MEFs (Fig 1A), although the causal mechanism is not known.

### Deletion of *Hottip* leads to reduced expression of posterior Hoxa *genes*

**Fig 3:**
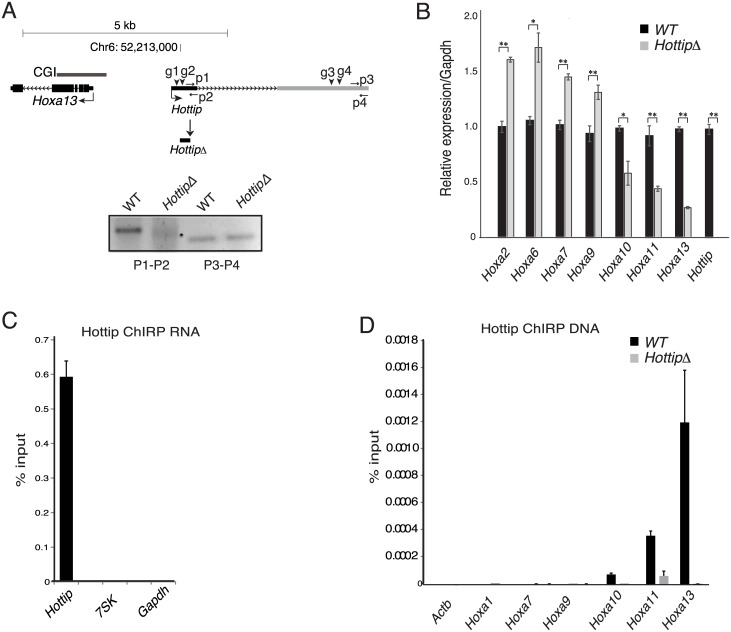
*Hottip* localizes to 5′ Hoxa genes and deletion of *Hottip* reduces *5*′ Hoxa expression. (A) Schematics showing the mouse *Hoxa13* and *Hottip* loci. The CpG Island (CGI) at the *Hoxa13* promoter is shown in a grey bar. Genome co-ordinates are from the mm9 assembly of the mouse genome. Guide RNA binding sites for deletion of *Hottip* are shown as arrow heads, primers used for genotyping are shown in arrows (p1 to p4). The deletion product of *Hottip* (*HottipΔ*) is shown below. Agarose gel image showing genotyping PCR, first two lanes are amplicons of primers (p1 and p2) within the deleted *Hottip* region, second two lanes are for amplicons from primers (p3 and p4) 3’ of deleted region. (B) Mean (± s.e.m) expression, assayed by RT-qPCR and normalized to *Gapdh*, of Hoxa genes and *Hottip*, in wild-type (black bars, WT), and *Hottip* knock out (gray bars, *HottipΔ*) limb mesenchymal cells, (n = 3 biological replicates). * p < 0.05, ** p <0.01. (C) RT-qPCR showing mean (± s.e.m) ± percentage (%) enrichment over input for *Hottip*, *7SK* and *Gapdh* RNAs from *Hottip* ChIRP pulldown from two experiments. (D) qPCR showing mean (± s.e.m) percentage (%) enrichment over input of ChIRPed DNA at promoters of *Actb*, *Hoxa1*, *Hoxa7*, *Hoxa9*, *Hoxa10*, *Hoxa11*, and *Hoxa13* from Hottip ChIRP experiments in wild type (black bars, WT) and *Hottip* knock out limb mesenchymal cells (grey bars, *HottipΔ*).

Most lncRNA depletion studies are done by si/sh RNA mediated knockdown, but the conclusions reached have often been different from those after genetic deletion of the loci encoding the lncRNAs[14–16]. We therefore used two pairs of guide RNAs with Cas9 nickase (Cas9n) to delete the gene body of *Hottip* (*HottipΔ*) in limb mesenchymal cells, leaving the *Hottip* promoter intact (Fig 3A). qRT-PCR of Hoxa genes showed a significant reduction in expression of *Hoxa13*, *a11* and *a10* in homozygous *HottipΔ* cells (Fig 3B). Consistent with *Psip1* and *Hottip* knock down studies (Fig 2B), expression of 3' Hoxa genes, such *Hoxa2*, *a6 and a7* increased in *HottipΔ* compared to WT cells. It is possible that effects on 3’ Hoxa genes are due to cross-regulation of Hox genes by Hox transcription factors [41].

### *Hottip* RNA is localized at posterior Hoxa genes

To find the direct genomic targets of *Hottip* lncRNA in limb cells, we performed chromatin isolation by RNA purification (ChIRP)[42] using 11 biotinylated antisense oligo pools covering the entire length of *Hottip*. qRT-PCR analysis of ChIRPed RNA showed specific enrichment for *Hottip* RNA (Fig 3C). qPCR analysis of *Hottip* ChIRPed DNA showed specific enrichment of *Hottip* RNA over the promoters of *Hoxa13*, and *a11* in WT cells. Analysis in *HottipΔ* cells confirmed the specificity of the *Hottip* ChIRP (Fig 3D). *Hottip* RNA was undetectable across more 3' Hoxa genes (*a9*, *a7*, *a1*), demonstrating that misregulation of 3' Hoxa genes in the absence of *Hottip* (Figs 2B and 3B) is a secondary event, which does not involve direct binding of *Hottip*.

### Induction of *Hottip* lncRNA is sufficient to activate posterior Hoxa genes

**Fig 4:**
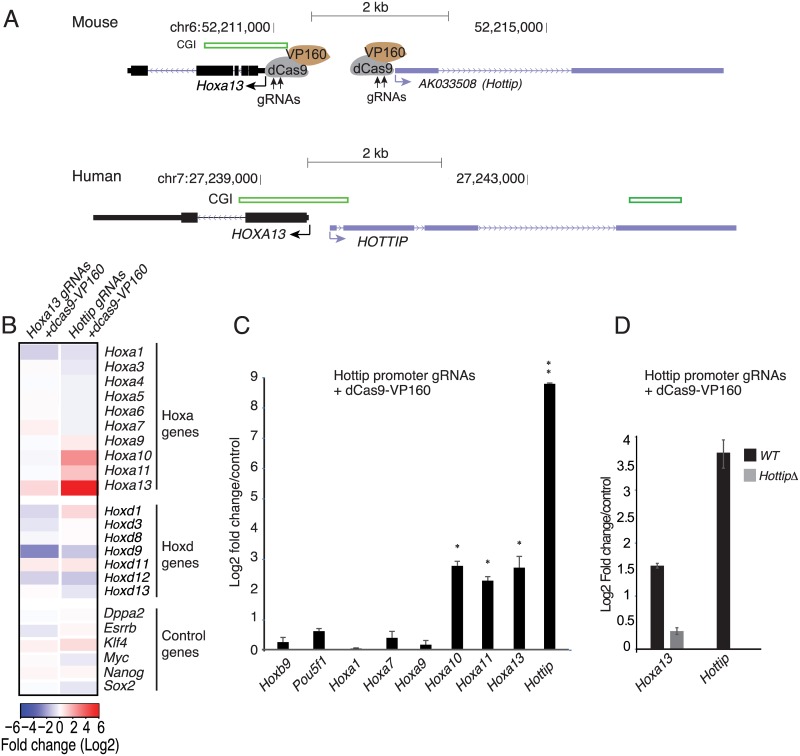
Artificial induction of *Hottip* is sufficient to activate 5’ Hoxa genes. (A) Schematics showing UCSC genomic coordinates of *Hottip*, *Hoxa13*, CpG Islands (CGI) in the mouse (top, mm9) and human (bottom, hg19) genomes. Schematics of guide RNA mediated recruitment of dCas9-VP160 to the *Hottip or Hoxa13* promoters is also shown. Direction of transcription is indicated as arrow marks. (B) Heat map showing the log2 mean fold change in expression of Hoxa, Hoxd and pluripotency associated genes (control genes) from expression microarray experiment, upon co-transfection of guide RNAs recognizing the *Hottip* promoter (*Hottip* gRNAs + dcas9-VP160). dCas9-VP160 was also co transfected with guide-RNAs recognizing *Hoxa13* promoter (*Hoxa13* gRNAs + dcas9-VP160) (n = 3 or 4 biological replicates). (C) Similar to (B) RT-qPCR data showing mean (± s.e.m) log2 fold change in expression of *Hottip*, *Hoxa13*, *a11*, *a10*, *a9*, *a7*, *a1*, *Pou5f1* and *Hoxb9* upon guide RNA mediated recruitment of dCas9-VP160 to the *Hottip* promoter (A) in mouse ES cells. Data were normalized to those from a dcas9 control (n = 3 biological replicates). (D) Similar to (C) mean log2 fold change in *Hottip and Hoxa13* expression in wild type ES cells co-transfected with guide-RNAs recognizing the *Hottip* promoter and dCas9-VP160 (Black bars, WT). *Hoxa13* expression in *Hottip* knock out limb mesenchymal cells is also shown (grey bar, *HottipΔ*). * p < 0.05, ** p <0.01 throughout.

It is possible that reduced expression of posterior Hox genes in *HottipΔ* cells is due to loss of *cis* -regulatory elements located within the deleted region, rather than loss of the *Hottip* RNA *per se*. *Hottip* is known to function at the site of its synthesis (*in cis*) and it fails to activate target genes when expressed ectopically from a retroviral construct [3]. Therefore, we synthetically activated endogenous *Hottip* in ES cells where *Hottip* and Hox gene clusters are repressed by polycomb complexes, to study the effect of lncRNA activation *in cis* or *trans*. We have previously shown that targeted recruitment of an ectopic activator (Vp16) to silent loci in murine ES cells (mESCs) can overcome this repression[43]. Unlike human *HOTTIP* which is transcribed bi-directionally from the *HOXA13* CpG island promoter (Fig 4A)[3], the mouse *Hottip* promoter is ~2 kb away from the TSS of *Hoxa13* which allowed us to recruit dcas9-Vp160 (Vp16 x10)[44–47] specifically to the promoters of either *Hottip* or *Hoxa13* (Fig 4A). CRISPR dCas9 mediated transcriptional activation has been shown to be ineffective when guides are targeted >1kb from TSS[44] suggesting that we should be able to direct activation specifically to *Hottip* or *Hoxa13* using this approach.

Agilent expression microarray and RT-qPCR analysis showed specific up-regulation of posterior (*a13*, *a11* and *a10*), but not anterior Hoxa and Hoxd genes upon dcas9-VP160 mediated *Hottip* activation in mESCs, relative to transfection with dCas9 recruitment alone (no VP160) (Fig 4B and 4C). In contrast, specific recruitment of dCas9-VP160 to the *Hoxa13* promoter led to up regulation of only *Hoxa13*, while expression of other Hoxa genes was unaltered (Fig 4B). Furthermore, recruitment of dCas9-VP160 to either *Hox13* or *Hottip* did not perturb the pluripotency network (Fig 4B) suggesting that the undifferentiated phenotype of the mESCs was not disrupted. Finally, recruitment of dCas9-VP160 to the *Hottip* promoter in *HottipΔ* 14fp cells led to only a modest upregulation of *Hoxa13* compared to WT cells (Fig 4D), pointing to the importance of full length *Hottip* RNA transcription in the regulation of Hoxa genes.

### *Hottip* RNA is indispensable for 5’ Hoxa expression

**Fig 5:**
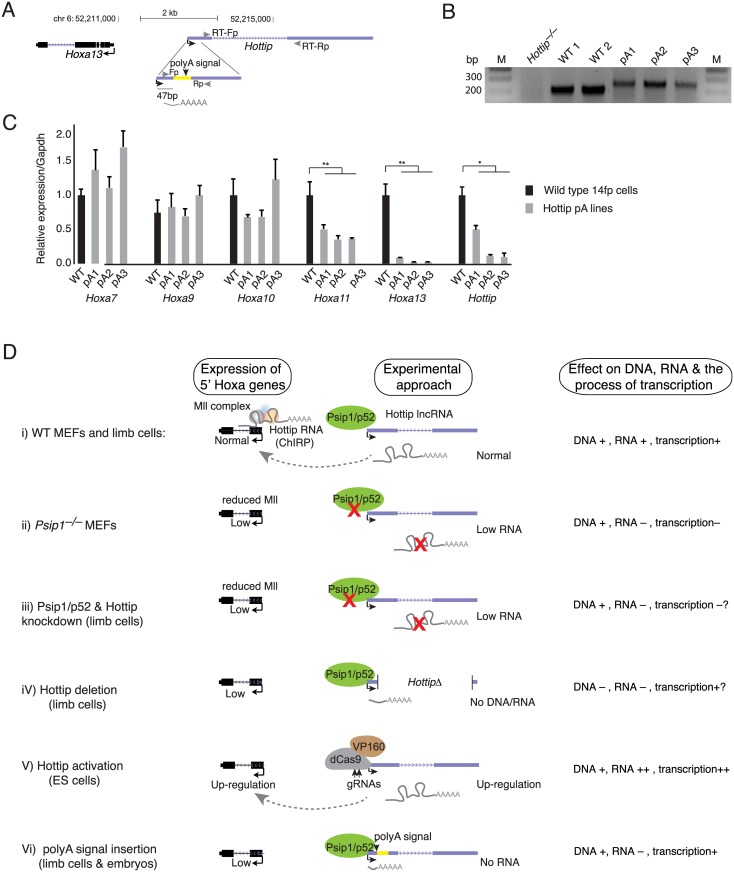
*Hottip* RNA is indispensable for 5’ Hoxa expression. (A) Schematics showing UCSC genomic coordinates of *Hottip* and *Hoxa13* in the mouse (mm9) genomes. Schematics of CRISPR mediated insertion of 49 bp synthetic polyA signal to ~47 bp downstream of *Hottip* transcription start site (TSS) is also shown (yellow). Primers used for genotyping and Sanger sequencing are shown in grey arrows, primers used for RT-qPCR are shown as RT-Fp and RT-Rp. (B) Genotyping PCR from the DNA isolated from the wild type (WT1, WT2) and polyA knock-in (pA1, pA2, and pA3) 14fp lines. (C) RT-qPCR data showing mean (± s.e.m of three technical replicates) fold change in expression of *Hottip*, *Hoxa13*, *a11*, *a10*, *a9* and *a7* in wild type (WT) and three polyA knock-in 14fp lines. * p < 0.05, ** p < 0.01. (D) Working model summarising the results from various experiments. Wild type cells with normal level of posterior Hoxa expression and occupancy of *Hottip* RNA at Hox genes (i). In *Psip1*^*–/–*^MEFs the expression levels of *Hottip*, posterior Hoxa genes and bound Mll levels are reduced (ii). Knockdown of Psip1/p52 or *Hottip* reduced expression of posterior Hoxa genes with corresponding reduction of Mll levels at these sites (iii). Deletion of *Hottip* leads to similar effect as depletion of *Hottip* by shRNAs (iV). Artificial activation of *Hottip* in mESCs leads to increased expression of target Hoxa genes (V). Premature termination of *Hottip* transcript leads to reduced expression of target Hoxa genes (Vi). The effects of each approach in changing the DNA element, lncRNA and the process of transcription are indicated (right).

To distinguish the requirement for the *Hottip* lncRNA molecule from the act of lncRNA transcription at the *Hottip* locus, for up-regulation of 5’ Hoxa genes, we used CRISPR-Cas9-mediated homologous recombination to insert a 49 bp synthetic polyadenylation (polyA) cassette[48] 47 bp downstream of the *Hottip* transcription start site (TSS) in 14fp cells (Fig 5A and 5B). Insertion of this polyA cassette should cause early cleavage of the nascent lncRNA transcript while preserving the promoter, and *cis* elements within the *Hottip* genomic locus. RT-qPCR analysis in three independent knockin lines and two wild-type (WT) clones demonstrated that spliced *Hottip* RNA was strongly reduced in all three polyA knockin lines (pA1, pA2 and pA3) compared to WT (Fig 5C). Importantly *Hoxa13* and *a11* mRNA levels were significantly reduced in all three pA lines compared to WT.

To verify this effect *in vivo*, we also injected Cas9 and guideRNAs into single cell zygotes to generate mouse embryos with a premature polyA signal inserted at *Hottip* (S1C Fig). Consistent with the results in the 14fp cell line, RT-qPCR analysis of polyA knockin 12.5 dpc embryo showed reduced expression of *Hottip*, *Hoxa13* and *Hoxa11* but not *Hoxc13 –*a posterior Hox gene from different chromosome (S1C Fig). This suggests that it is the full length Hottip RNA itself that is involved in *Hoxa13/a11* regulation.

## Discussion

Our findings are compatible with a model in which *Hottip* lncRNA regulates posterior *Hoxa* gene transcription *in cis* (Fig 5D)—likely through an Mll complex. We have previously shown that the longer p75 isoform of Psip1 binds directly to Mll through its MLL or integrase binding domain (IBD) and recruits Mll to active Hox clusters[35]. Here we have demonstrated that the shorter isoform Psip1/p52 –which lacks the C-terminal Mll1 binding domain of p75—controls posterior Hoxa genes by activating the expression of *Hottip* lncRNA, a mechanism quite distinct from p75. We show that the *Hottip* lncRNA itself is required to maintain active expression of 5’ Hoxa genes, possibly by maintaining a stable Mll1 complex at the 5’ end of Hoxa gene cluster. The mechanism through which *Hottip* RNA specifically localizes to 5’ Hoxa genes *in cis* is not clear and needs further investigation.

By inserting a polyadenylation cassette at the 5’ end of *Hottip*, we show the importance of *Hottip* RNA for *Hoxa13* expression in both cell lines and *in vivo* in mouse embryogenesis. Similarly, *Hottip* is upregulated in several cancers where its expression also correlates with increased *Hoxa13* [49–51]. Recently, two micro RNAs miR-192, miR-204 have been demonstrated to post-transcriptionally silence the *HOTTIP* lncRNA, leading to the reduced viability of hepatocellular carcinoma (HCC) cells[52], further validating a role for this lncRNA molecule. Further studies are needed to understand whether human *HOTTIP/HOXA13* are regulated by PSIP1, and the role of PSIP1 and *HOTTIP* in oncogenesis.

Noncoding transcription at enhancer elements has been associated with enhancer activity[53,54]. However, most enhancer RNAs (eRNAs) are degraded by exosomes, suggesting that at distal regulatory elements the act of transcription itself could be sufficient for the enhancer activity [55–57]. An enhancer -like function of lncRNAs has been demonstrated in some cases including *HOTTIP*[3,58]. However, an increasing body of evidence suggests that the function of many lncRNA genes in regulating genes *in cis* does not require the lncRNA molecule itself. Instead their effect is mediated by enhancer-like activity of underlying DNA elements in the lncRNA locus, the act of transcription and/or splicing of lncRNAs[59–62].

The recent controversies in the lncRNA field demand thorough investigations to distinguish the role of lncRNA molecules from enhancer- like function of the DNA elements which encode them, and from the process of transcription and splicing of these loci. Our studies presented here show how these facets of lncRNA regulation and function can be dissected at one well-studied lncRNA locus. With the ever increasing number of lncRNAs annotated in genomes using high-throughput sequencing technologies, the list of these transcripts with unknown mechanisms of upstream transcriptional regulation and downstream functional mechanism is growing and there will be the need to develop more high-throughput methods for the rigorous testing of lncRNA function and mechanism of action.

## Methods

### Ethics statement

Cervical dislocation was used as a euthanasia method and all mouse experiments were performed under the Animals (Scientific Procedures) Act 1986' and were approved by the University of Edinburgh ethical committee (TR-38-16) and performed under UK Home Office license number PPL 60/4418.

### Cell lines

*Psip1*^*–/–*^and its corresponding WT MEFs[35,63] were a kind gift from Prof. Alan Engelman (Dana-Farber Cancer Institute, USA). Limb mesenchymal cells (14fp) isolated from the posterior mesenchyme of E11.5 mouse embryos from an Immortomouse (H-2kb-tsA58) × CD1 cross, are as previously described[39] and were a gift from Robert Hill (MRC Human Genetics Unit, University of Edinburgh). mES cells (E14) were cultured as previously described[64]. Psip1/p52 rescue experiment in *Psip*^*–/–*^MEFs is previously described[35].

### shRNA knockdown

Lentiviral shRNAs (pLKO.1 vectors) targeting Psip1/p52, Psip1/p75 and *Hottip* (S1 Table) were transduced as described by the manufacturer (Sigma Aldrich). Expression of p52 was rescued by transiently transfecting a shRNA-resistant p52 cDNA[35].

### RT-qPCR

Reverse transcription followed by quantitative PCR (RT-qPCR) was performed as described previously[35]. Briefly, RNA was treated with Turbo DNA Free kit (ThermoFisher Scientific) and cDNA was prepared using superscript II reverse transcriptase (ThermoFisher Scientific) using random primers. All qPCRs were performed with three biological or technical replicates in a LightCyler 480 (LC480, Roche) or CFX96 (Biorad), and the data was normalized to *Gapdh*. Details of the oligos are given in the S2 Table.

### Whole mount RNA *in situ* on mouse embryos

RNA in situ hybridization for *mHottip* in 10.5 dpc mouse embryos were performed as previously described[65]. Details of oligos used to PCR amplify the *Hottip* cDNA including T7 (sense) and T3 (Antisense) promoter sequences are given in S3 Table.

### ChIP, antibodies and data analysis

ChIP was performed as described previously[35], using antibodies for Mll1 (Active Motif 61295, 61296), ChIP DNA was hybridized to a custom *Hox* array and data was normalized as described previously[35].

### Chromatin Isolation by RNA Purification (ChIRP)

Anti-sense oligo probes tiling the mouse *Hottip* RNA were designed using the web tool from Stellaris FISH Probe Designer (https://www.biosearchtech.com/support/education/stellaris-rna-fish) Biosearch Technologies, CA, USA). Eleven biotinylated oligos were synthesized by Sigma-Aldrich (S6 Table). ChIRP was performed in limb mesenchymal cells as described previously[42]. RNA was isolated from 20% of the ChIRPed beads and used for RT-qPCR for *Hottip*, *7sk* and *Gapdh* specific primers and rest of the sample was used to purify DNA and perform qPCR for Hoxa genes. Primer details are given in S2 Table.

### CRISPR mediated deletion of *Hottip*

Guide RNAs were designed using the Zhang laboratory web tool (http://crispr.mit.edu). Paired guide RNAs (gRNAs) (S4 Table) were designed to target the murine *Hottip* genomic locus ~50bp beyond the TSS and before the transcription end site (Fig 3A). gRNAs were cloned into the D10A nickase mutant version of cas9 (cas9n) containing pSpCas9n(BB)-2A-GFP (PX461)[66]. A pool of four gRNA containing plasmids were transfected into mouse limb-bud mesenchymal cells (14fp) using FuGENE HD transfection reagent and FACS sorted 48 hours after transfection for GFP^+^ cells. Homozygous deletion of *Hottip* was confirmed by PCR and Sanger sequencing, primers used are given in S2 Table.

### dCas9-mediated activation of *Hottip* and *Hoxa13* in ES cells

Five guide RNA plasmid pools targeting the promoters of *Hottip* and *Hoxa13* (S5 Table) were designed as above and cloned into pSLQ1371[56,67]. These gRNA plasmids encoding mCherry and puromycin resistance were co-transfected with a plasmid encoding dCas9-VP160 (pAC94-pmax-dCas9VP160-2A-puro, Addgene plasmid number 48226) [47]. 24hrs after transfection, transfected mES cells were selected by addition of 2μg/ml puromycin for another 24 hrs. RNA was extracted 48hrs after transfection, RT-qPCR was performed as described above. Microarray gene expression analysis performed according to the manufacturer’s protocol (Agilent Technologies). Plasmids containing non-targeting guide RNAs and dCas9 alone (dcas9Δ) served as controls.

### dCas9-mediated activation of *Hottip* in 14fp cells

Wild type and *HottipΔ* 14fp cells were transfected with *Hottip* gRNA plasmid pools similar to ES cells and FACS sorted for mCherry positive cells 24 hrs after transfection. Transfected mCherry positive cells were seeded to cell culture flasks to recover for another 24 hrs, 48 hrs after transfection cells were harvested and RNA was isolated using Trizol and RT-qPCR was performed.

### CRISPR mediated insertion of polyA sites into *Hottip*

*HottipΔ* 5'guide 1 oligos (1 & 2 in S4 Table) were cloned into pSpCas9(BB)-2A-Puro (PX459) V2.0 (a gift from Feng Zhang (Addgene plasmid 62988)), which is designed to insert a synthetic polyA signal sequence into the *Hottip* genomic locus 47 bp after the TSS (Fig 5A). gRNA containing plasmids were co-transfected along, with a repair template (S7 Table) synthesized as a 199bp single-stranded Ultramer oligo (IDT) bearing the desired sequence change, into limb cells using lipofectamine 2000 transfection reagent. 24 hours after transfection puromycin resistant cells were selected for another 48 hours and plated at 2500 cells/100mm plates. On day 10 colonies were picked and plated in duplicate into 96 well plates. Genomic PCR (Fig 5B) and Sanger sequencing confirmed three polyA knockin (pA) clonal lines with homozygous insertions of the polyA cassette into exon 1 of *Hottip*. Primers used for genotyping PCR sequencing and RT PCR are given in S2 Table.

### CRISPR mediated insertion of polyA sites into *Hottip* in mouse embryo

To generate mouse embryos with a premature transcriptional termination signal (polyA signal) at the *Hottip* locus, single cell mouse zygotes were injected with Cas9 mRNA (50ng/ul), gRNA (25ng/ul) and repair template DNA (75ng/ul) (S7 Table). The embryos were later harvested for analysis at 12.5 dpc stage of embryonic development, tail tips were used to genotype the embryos by PCR and Sanger sequencing (S1C and S1D Fig). Total RNA was isolated from a PolyA knockin embryo and two litter mate wild types using Trizol and reverse transcribed using Superscript II and qPCR was performed using iTaq universal SYBR green supermix (Biorad).

## Supporting information

S1 Fig Whole mount *in situ* hybridization and PolyA insertion data from mouse embryos. (A) Whole mount RNA in situ hybridization of *Hottip* in 10.5d embryo (right). Image from Psip1 RNA in situ hybridization for 11.5d embryos from Embrys resource (left) http://www.emouseatlas.org/emagewebapp/pages/emage_general_query_result.jsf. (B) Similar to Fig 2B mean (± s.e.m) expression, assayed by RT-qPCR and normalized to *Gapdh*, of *Hoxa* genes, along with *Psip1/p52*, *Psip1/p75*, and *Hottip* RNA, in limb cells transduced with independent shRNAs (sh2’s in S1 Table) targeting p52 (red bars, p52 sh2) p75 (green bars, p75 sh2) and Hottip (black bars, Hottip sh2) relative to cells transduced with a mammalian non-targeting sh RNA (Grey bars, control) (n = 3 biological replicates, p value * <0.05, ** <0.01). (C) Similar to Fig 5B, genotyping PCR from the DNA isolated from the wild type (WT1, WT2) and polyA knockin (Hottip pA1) 12.5 dpc embryo. (D) Similar to Fig 5A, illustration showing polyA insertion site within *Hottip* gene (yellow), Sanger sequencing data confirms 49 bp polyA signal sequence insertion (highlighted in yellow) and flanking Hottip sequence. (E) Similar to Fig 5A and 5C, RT-qPCR data showing mean (± s.e.m of three technical replicates) and normalized to *Gapdh*, fold change in expression of *Hottip*, *Hoxa13*, *a11*, *a10*, *a9* and *Hoxc13* in two wild type (WT1 and WT2) and one polyA knock-in 12.5 dpc whole embryo at Hottip locus.
(PDF)

S1 Table ShRNA sequences or TRC numbers. (DOCX)

S2 Table List of primers used for RT-qPCR, and genotyping PCR. (DOCX)

S3 Table Oligos used to PCR amplify *Hottip* cDNA to prepare sense and antisense probes used in whole mount *in situ* (S1 Fig). (DOCX)

S4 Table Oligos used to clone guides to pX461. (DOCX)

S5 Table Forward oligos used to clone guideRNA (gRNA) sequences to pSLQ plasmids, target sequences of the sgRNAs (Fig 4) are shown in red. (DOCX)

S6 Table Oligos used in ChIRP experiment. (DOCX)

S7 Table PolyA repair template. Nucleotide sequence of single stranded oligonucleotide used as homology directed repair templated used for insertion of a synthetic polyA signal sequence to hottip locus. 75 base nucleotide homology arms are shaded in grey, 49base polyA signal is shaded in yellow. GuideRNA binding site is in red, PAM site is in blue.
(DOCX)
