# Supplementary figures and images for: Psip1/p52 regulates posterior Hoxa genes through activation of lncRNA Hottip
Source: PLoS Genet. 2017 Apr 6;13(4):e1006677. doi: 10.1371/journal.pgen.1006677 (PMC5383017; doi:10.1371/journal.pgen.1006677)

A

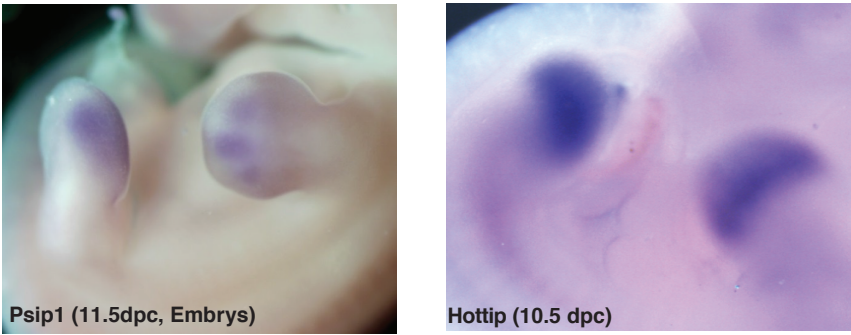

B

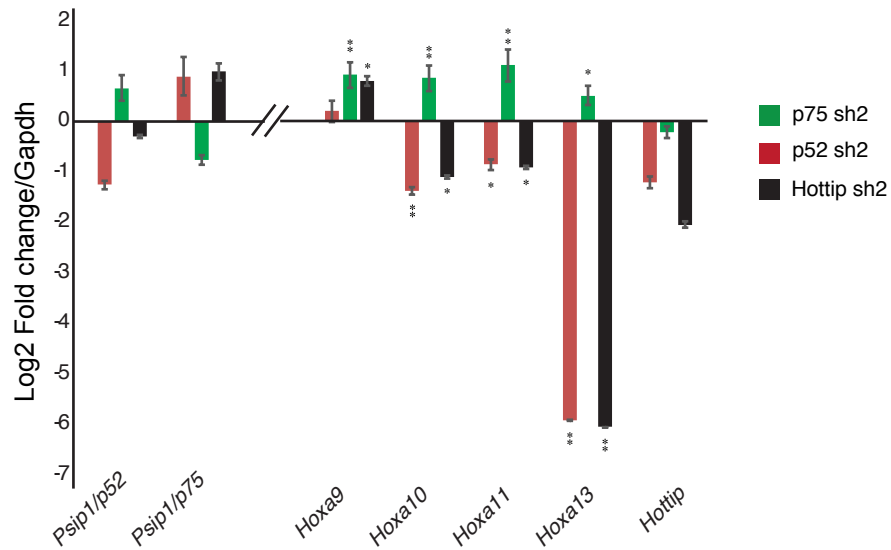

C

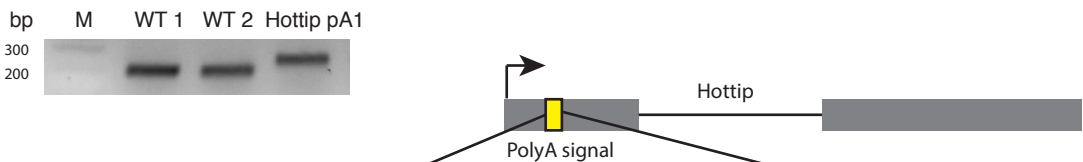

D

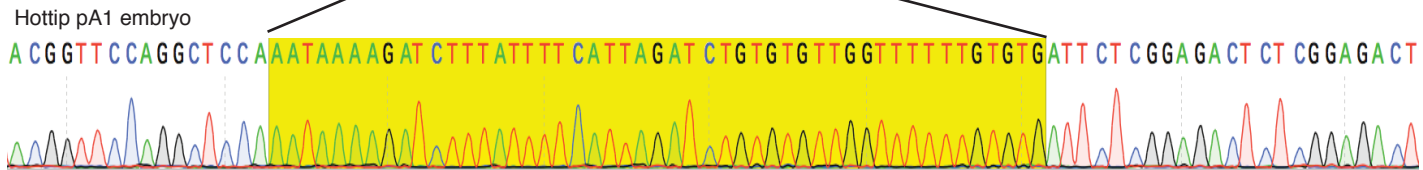

E

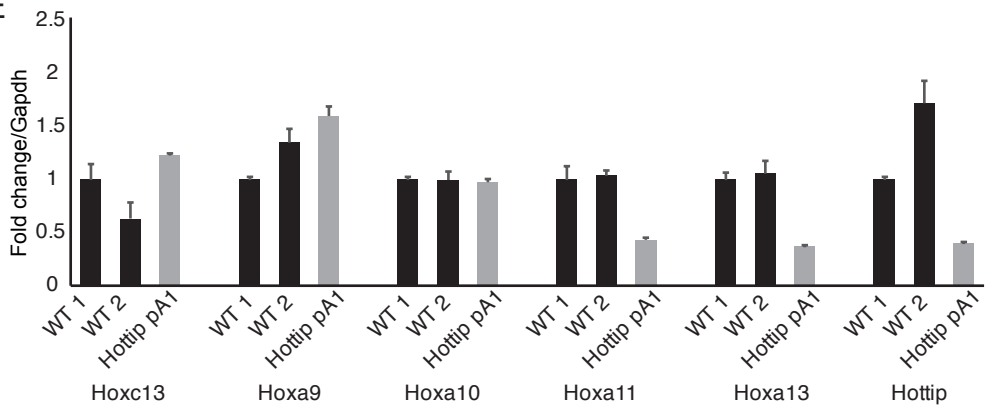

S1 Fig

Supplement: S1 Fig — (A) Whole mount RNA in situ hybridization of Hottip in 10.5d embryo (right). Image from Psip1 RNA in situ hybridization for 11.5d embryos from Embrys resource (left) http://www.emouseatlas.org/emagewebapp/pages/emage_general_query_result.jsf. (B) Similar to Fig 2B mean (± s.e.m) expression, assayed by RT-qPCR and normalized to Gapdh, of Hoxa genes, along with Psip1/p52, Psip1/p75, and Hottip RNA, in limb cells transduced with independent shRNAs (sh2’s in S1 Table) targeting p52 (red bars, p52 sh2) p75 (green bars, p75 sh2) and Hottip (black bars, Hottip sh2) relative to cells transduced with a mammalian non-targeting sh RNA (Grey bars, control) (n = 3 biological replicates, p value * <0.05, ** <0.01). (C) Similar to Fig 5B, genotyping PCR from the DNA isolated from the wild type (WT1, WT2) and polyA knockin (Hottip pA1) 12.5 dpc embryo. (D) Similar to Fig 5A, illustration showing polyA insertion site within Hottip gene (yellow), Sanger sequencing data confirms 49 bp polyA signal sequence insertion (highlighted in yellow) and flanking Hottip sequence. (E) Similar to Fig 5A and 5C, RT-qPCR data showing mean (± s.e.m of three technical replicates) and normalized to Gapdh, fold change in expression of Hottip, Hoxa13, a11, a10, a9 and Hoxc13 in two wild type (WT1 and WT2) and one polyA knock-in 12.5 dpc whole embryo at Hottip locus. (PDF) [file pgen.1006677.s001.pdf]
