# Supplementary material for: Psip1/p52 regulates posterior Hoxa genes through activation of lncRNA Hottip
Source: PLoS Genet. 2017 Apr 6;13(4):e1006677. doi: 10.1371/journal.pgen.1006677 (PMC5383017; doi:10.1371/journal.pgen.1006677)
Supplement: S1 Table — (DOCX) [file pgen.1006677.s002.docx]

**S1 Table:** ShRNA sequences or TRC numbers

| **Target RNA** | **Name** | **Sequence (5-3’)** | **Target region** |
| --- | --- | --- | --- |
| Psip1/p75 | p75 Sh1 | TRCN0000012116 (Sigma Aldrich) | CDS |
| Psip1/p75 | p75 Sh2 | TRCN0000012113 (Sigma Aldrich) | 3’ UTR |
| Psip1/p52 | p52 Sh1 | TTTGGGCTCAAAGCATTAATC | 3’ UTR |
| Psip1/p52 | p52 Sh2 | CAGACTCATTGGACTGAATTT | 3’ UTR |
| Hottip | Hottip Sh1 | CGGGCTTTATATCAACAAATA | NA |
| Hottip | Hottip Sh2 | GGGACCGAATTCCTGTAAAT | NA |
