# Supplementary material for: Psip1/p52 regulates posterior Hoxa genes through activation of lncRNA Hottip
Source: PLoS Genet. 2017 Apr 6;13(4):e1006677. doi: 10.1371/journal.pgen.1006677 (PMC5383017; doi:10.1371/journal.pgen.1006677)
Supplement: S2 Table — (DOCX) [file pgen.1006677.s003.docx]

**S2 Table:** List of primers used for RT-qPCR

| **RTq-PCR** | **Forward (5'-3')** | **Reverse (5'-3')** |
| --- | --- | --- |
| Hoxa2 | CTGCCTCGGCCACAAAGAATCC | AGCTGAGTGTTGGTGTACGCG |
| Hoxa6 | CACCGACCGGAAGTACACAAG | TGTCTGGTAGCGCGTGTAGGTC |
| Hoxa7 | AAGCCAGTTTCCGCATCTACC | GTAGCGGTTGAAATGGAATTCC |
| Hoxa9 | AGAAAAACAACCCAGCGAAG | CAGTTCCAGCGTCTGGTGT |
| Hoxa10 | GTGTCAAGTCCTGAATGGGC | AGAGAAACCAGGCCTGGACT |
| Hoxa11 | CACTGAGGACAAGGCCGGTG | TTGAGCATGCGGGACAGTTG |
| Hoxa13 | CACCTCTGGAAGTCCACTCT | TCTCAGAGAGGTTTGTCGTG |
| Hottip spliced | GGCTCCAAAAATGGTGAGAA | GAGGGTGATGCAGTTGGAGT |
| Hottip exon2 | TAGCGGGTTTGCGTCTAAGT | CCAGATGGGGAGTGTCCTTA |
| Hoxb9 | AGGAAGCGAGGACAAAGAGAGG | CTTGTCTCTCACTCAGATTGAG |
| Pou5f1 | CGAGAACAATGAGAACCTTC | CCTTCTCTAGCCCAAGCTGAT |
| Psip1/p52 | TCCATTTGTTCCTCCTGCTT | GGTGTGCCCTTGACAGTCTT |
| Psip1/p75 | ACAATGCAGCAAGCTCAGA | ACTGACTTTGAACCGCCGTA |
| 7SK | GACATCTGTCACCCCATTGA | GCCTCATTTGGATGTGTCTG |
| Gapdh | TGGTGAAGGTCGGTGTGAACG | TGAGTGGAGTCATACTGG |
| **ChIRP-qPCR** |  |  |
| Actb_Pro | CCTCGATGCTGACCCTCATCC | GACACTGCCCCATTCAATGTCTC |
| Hoxa1_Pro | CTACACTGAGGAGCGCCGGGGATT | CCGCGCAGGATTGGAAAGTTGTAA |
| Hoxa7_Pro | GAGAGGTGGGCAAAGAGTGG | CCGACAACCTCATACCTATTCCTG |
| Hoxa10_Pro | ATGTTTGAGGCCGTACTGGT | CTTCCCAAAGTGGCTGGTAA |
| Hoxa11_Pro | AGCCCAATGATGGATTTTGA | GAAGGGAGGCTGGAGAAATC |
| Hoxa13_Pro | TAGAGCTCGGCTCCTCTCAG | CCAAATTGTCCCTGATGGTT |
| Hottip_Δ PCR & sequencing | TGCCCCGAGTTTACCTTCTT | CTCTGTGTCCTAGGCCATGT |
| Hottip_ΔRT-PCR | CTCAACAGTGACAAAGAGACGAA | GCATTTGCTTAACGTGAAAATG |
| Hottip pA screening | GAAGTACGGTTCCAGGCTC | AGCCAGAAGCCAGATAGCAA |
