# Supplementary material for: Psip1/p52 regulates posterior Hoxa genes through activation of lncRNA Hottip
Source: PLoS Genet. 2017 Apr 6;13(4):e1006677. doi: 10.1371/journal.pgen.1006677 (PMC5383017; doi:10.1371/journal.pgen.1006677)
Supplement: S3 Table — (DOCX) [file pgen.1006677.s004.docx]

**S3 Table:** Oligos used to PCR amplify Hottip cDNA to prepare sense and antisense probes for whole mount *in situ* (S1 Fig)

| **Primer** | **Sequence (5-3’)** |
| --- | --- |
| Hottip T7 sense FP | GATCCGTAATACGACTCACTATAGGGTACGGTTCCAGGCTCCAATT |
| Hottip T3 antisence RP | AGTCGCGCGAAATTAACCCTCACTAAAGGCAAACAAAACATTCGTGCTT |
