# Supplementary material for: Psip1/p52 regulates posterior Hoxa genes through activation of lncRNA Hottip
Source: PLoS Genet. 2017 Apr 6;13(4):e1006677. doi: 10.1371/journal.pgen.1006677 (PMC5383017; doi:10.1371/journal.pgen.1006677)
Supplement: S4 Table — (DOCX) [file pgen.1006677.s005.docx]

**S4 Table:** Oligos used to clone guides to pX461

| **Hottip deletion guides** | **Sequence (5-3’)** |
| --- | --- |
| 1. Hottip5’guide 1top | CACCGCTCCGAGAGTCTCCGAGAAT |
| 1. Hottip5’ guide 1bottom | AAACATTCTCGGAGACTCTCGGAGC |
| 1. Hottip5’ guide 2top | CACCGCTCGAGGGCAGTTTACATAC |
| 1. Hottip5’ guide 2bottom | AAACGTATGTAAACTGCCCTCGAGC |
| 1. Hottip3’ guide 1top | CACCGGGCCCACTTACTCAGTTTCC |
| 1. Hottip3’ guide 1bottom | AAACGGAAACTGAGTAAGTGGGCCC |
| 1. Hottip3’ guide 2top | CACCGGCACTCCCTCCCGCTTTGTAC |
| 1. Hottip3’ guide 2bottom | AAACGTACAAAGCGGGAGGGAGTGCC |
