# Supplementary material for: Psip1/p52 regulates posterior Hoxa genes through activation of lncRNA Hottip
Source: PLoS Genet. 2017 Apr 6;13(4):e1006677. doi: 10.1371/journal.pgen.1006677 (PMC5383017; doi:10.1371/journal.pgen.1006677)
Supplement: S5 Table — (DOCX) [file pgen.1006677.s006.docx]

**S5 Table:** Forward oligos used to clone guideRNA (gRNA) sequences to pSLQ plasmids, target sequences of the sgRNAs (Figure 4) are shown in red

| **gRNA name** | **Oligo Sequence** |
| --- | --- |
| mHottip_pro_sg1 | ggagaaCCACCTTGTTGGGCAGTAAGAAGGTAAACTCGGTTTAAGAGCTATGCTGGAAACAGCA |
| mHottip_pro_sg2 | ggagaaCCACCTTGTTGGTCTCCTGACTTTAGCGGTCCGTTTAAGAGCTATGCTGGAAACAGCA |
| mHottip_pro_sg3 | ggagaaCCACCTTGTTGGTACCCAGGACCGCTAAAGTCGTTTAAGAGCTATGCTGGAAACAGCA |
| mHottip_pro_sg4 | ggagaaCCACCTTGTTGGGGATCAGGGAAGGTTTTATTGTTTAAGAGCTATGCTGGAAACAGCA |
| mHottip_pro_sg5 | ggagaaCCACCTTGTTGGGGGATCAGGGAAGGTTTTATGTTTAAGAGCTATGCTGGAAACAGCA |
|  |  |
| Hoxa13_Pro_sg1 | ggagaaCCACCTTGTTGGTCCCGCTTTGCATACGCCGGGTTTAAGAGCTATGCTGGAAACAGCA |
| Hoxa13_Pro_sg2 | ggagaaCCACCTTGTTGGCATACGCCGGGGGCGGGCCGGTTTAAGAGCTATGCTGGAAACAGCA |
| Hoxa13_Pro_sg3 | ggagaaCCACCTTGTTGGCGCTTTGCATACGCCGGGGGGTTTAAGAGCTATGCTGGAAACAGCA |
| Hoxa13_Pro_sg4 | ggagaaCCACCTTGTTGGCGGGCCGGGGGCGGGCCAATGTTTAAGAGCTATGCTGGAAACAGCA |
| Hoxa13_Pro_sg5 | ggagaaCCACCTTGTTGGCGGGCCAATGGGCGGCCGCCGTTTAAGAGCTATGCTGGAAACAGCA |
