# Supplementary material for: Psip1/p52 regulates posterior Hoxa genes through activation of lncRNA Hottip
Source: PLoS Genet. 2017 Apr 6;13(4):e1006677. doi: 10.1371/journal.pgen.1006677 (PMC5383017; doi:10.1371/journal.pgen.1006677)
Supplement: S6 Table — (DOCX) [file pgen.1006677.s007.docx]

**S6 Table:** Oligos used in ChIRP experiment

| **Oligo name** | **Oligo sequence (5'-3')** |
| --- | --- |
| Hottip ChiRP oligo 1 | CTCTTTGTCACTGTTGAGTT Biotin tag |
| Hottip ChiRP oligo 2 | AGAAAGAACCAGTCCAGCC Biotin tag |
| Hottip ChiRP oligo 3 | GACTTTGGCAGTCTGTAGAG Biotin tag |
| Hottip ChiRP oligo 4 | GCCATTAGAACACGGAGAGA Biotin tag |
| Hottip ChiRP oligo 5 | AGCATCAACCAGGTTGAGAA Biotin tag |
| Hottip ChiRP oligo 6 | AGAATTCACAGCCAGATGGG Biotin tag |
| Hottip ChiRP oligo 7 | TTTGACAAGGTGATTTTCCC Biotin tag |
| Hottip ChiRP oligo 8 | TAGTCCCACAGACAAAGGAC Biotin tag |
| Hottip ChiRP oligo 9 | AATGGCCCACTTACTCAGTT Biotin tag |
| Hottip ChiRP oligo 10 | CAGTTCAATACCTGGGATAT Biotin tag |
| Hottip ChiRP oligo 11 | ATCAAGTTAGGACACAGGGC Biotin tag |
