# Supplementary material for: Psip1/p52 regulates posterior Hoxa genes through activation of lncRNA Hottip
Source: PLoS Genet. 2017 Apr 6;13(4):e1006677. doi: 10.1371/journal.pgen.1006677 (PMC5383017; doi:10.1371/journal.pgen.1006677)
Supplement: S7 Table — Nucleotide sequence of single stranded oligonucleotide used as homology directed repair templated used for insertion of a synthetic polyA signal sequence to hottip locus. 75 base nucleotide homology arms are shaded in grey, 49base polyA signal is shaded in yellow. GuideRNA binding site is in red, PAM site is in blue. (DOCX) [file pgen.1006677.s008.docx]

**S7 Table:** PolyA repair template

CCACTCCTTCCTCCCTCTAAGTGGCATTGTAAAACTCAACAGTGACAAAGAGACGAAGTACGGTTCCAGGCTCCAAATAAAAGATCTTTATTTTCATTAGATCTGTGTGTTGGTTTTTTGTGTGATTCTCGGAGACTCTCGGAGACTCGAGGGCAGTTTACATACAAGTCAGACACGCTGGAGGCCAAGGTCAAGTTGA
